# A Prospective study of the association between weight changes and self-rated health

**DOI:** 10.1186/1472-6874-8-13

**Published:** 2008-08-08

**Authors:** Mette K Simonsen, Yrsa A Hundrup, Morten Grønbæk, Berit L Heitmann

**Affiliations:** 1 Research Unit for Dietary Studies, Institute of Preventive Medicine, Centre for Health and Society, Copenhagen, Denmark; 2 Danish Nurse Cohort Study, Research Centre for Prevention and Health (RCPH), Glostrup, Denmark; 3 National Institute of Public Health, Copenhagen, Denmark

## Abstract

**Background:**

Obesity and self-rated health (SRH) are strong predictors of morbidity and mortality but their interrelation is sparsely studied. The aim of this study was to analyse the association between weight changes and changes in SRH among women. We also examined if poor SRH at baseline was associated with later weight gain.

**Methods:**

The Danish Nurse Cohort Study is a prospective population study (1993–1999) and comprises 13,684 female nurses aged 44 to 69 years. Logistic regression analyses were used to examine the association between weight changes and changes in SRH.

**Results:**

Women who gained weight during the study period had higher odds of reporting poorer self-rated health (Odds Ratio (OR): 1.18, 95% CI: 1.04–1.35). Weight loss among overweight women, did not result in an increase in self-rated health ratings, in fully adjusted analyses (0.96 (95% CI: 0.76–1.23). Poor self-rated health combined with normal weight at first examination was associated with higher odds of later weight gain (OR: 1.29, 95% CI: 1.10–1.51).

**Conclusion:**

Weight changes may result in lower SRH. Further, poor self-rated health at baseline seems to predict an increase in weight, among women without any longstanding chronic diseases. Future obesity prevention may focus on normal weight individuals with poor SRH.

## Background

With more than one billion overweight adults globally, obesity has reached epidemic proportions and the World Health Organization (WHO) estimates obesity to be one of our times greatest threat to Public Health [1,2]. Overweight or obesity (Body Mass Index (BMI) ≥ 25 kg/m^2^) increases the risk of many diseases: hypertension, type 2 diabetes, cardiovascular diseases, muscular- and skeleton diseases, and respiratory problems, among others [3]. Furthermore, obese experience social stigmatisation, rejection from the labour market, depressions [4,5] and poor self-rated health [6] more often than normal weight individuals. In cross-sectional studies a J-shaped association has been found between weight and self-rated health (SRH), indicating that underweight and overweight, in particular, have negative influences on self-reported health ratings [7,8]. Thus, underlying diseases among underweight individuals may explain this J-shaped association. Studies have found several physiological advantages of weight loss among overweight individuals [9-11]. A newly published review and meta-analysis find that individuals who enter weight loss treatments emerge with less depression and greater self-esteem [12]. Similar results are found in studies examining the association between weight loss and health-related quality of life [13,14]. In agreement with these findings, weight gain has been shown to be associated with decreased well-being [15]. Therefore, Health Authorities usually recommend weight loss when BMI ≥ 27 [16]. Also, in the general public opinion, weight loss is associated with better health-related quality of life, and weight gain is associated with poor health and lower quality of life. However, prospective studies examining the association between weight changes and mortality often find contradictions to the conventional wisdom, as several of these studies find that weight loss increases mortality risk [16-18].

The purpose of this study was to examine the association between weight change and change in SRH over a 6-year period, and to analyse whether weight change had an effect on SRH. Furthermore, we wanted to examine if poor SRH at baseline was associated with later weight loss or with weight gain. SRH is a strong predictor of mortality [19], and may therefore be a useful outcome-measure, in public health prevention [20]. In the present study we hypothesized that overweight women who become normal weight, would rate their health better than women who were constantly overweight. We also wanted to analyse whether individuals who rated their health as poor at baseline, had an increased risk of later weight loss, indicating that obesity and poor SRH could be associated by reversed causality.

As a primary research question we wanted to examine if weight loss among overweight women had a negative effect on SRH (hypothesis 1). As a secondary research question we wanted to examine if poor SRH at baseline would lower the odds of gaining weight due to underlying diseases (hypothesis 2).

## Methods

### Study population

**Figure 1:**
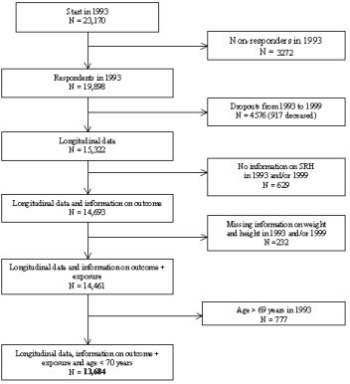
The number of non-responders and dropouts due to missing information.

The Danish Nurse Cohort Study was established in 1993 by mailing a questionnaire to all female nurses above the age of 44 years, who were members of the Danish Nursing Council and who lived in Denmark. In all 23,170 women, of whom 19,898 (86%) responded. The investigation was reported to the Registry Supervisory Committee (1993-1110-1151) and the Ethics Committee j.nr.(KF)01-103/93. Both committees approved the study, and the Danish Nursing Council allowed us to use the membership database. The cohort was re-examined in 1999. In the present study, women who received and returned a questionnaire in both 1993 and 1999 were included. In all 15,322 (77%) responded [21]. Dropouts in 1993 and 1999, participants with no information on SRH, weight and height in 1993 and/or 1999 and participants above the age of 69 years at baseline were excluded in order to avoid age induced unintentional weight loss [22,23]. Also, the impact of weight on mortality differs between women who are younger and older than 65 years [24]. Women who were lost to follow-up were more likely to be underweight and to rate their health poorer than the responders. In total, the study population comprised 13,684 participants. Figure 1 shows the number of non-responders and dropouts due to missing information [see Figure 1].

### Variables included in the analysis

#### Exposure variables

When we estimated the association between changes in self-reported BMI-categories and changes in SRH we used changes of BMI as exposure variable. When the exposure variable was BMI-categories, we used the WHO definition of underweight, normal weight, overweight and obesity. BMI categories, defined by the World Health Organization [1] were used to discriminate between underweight (BMI < 18.5), normal weight (18.5 ≤ BMI < 25.0), overweight (25.0 ≤ BMI < 30.0) and obese (BMI ≥ 30.0).

#### Outcome variables

The outcome variable was changes in SRH from 1993 to 1999. SRH is defined as a person's assessment of own health. The women in this study were asked, "How would you rate your health in general?" and the response alternatives were: Very good, good, fair, bad, very bad. Between all five original SRH responses the change category in SRH had a total of 9 possible steps. An increase or a decrease in SRH was defined as a move, up towards better health (4 possible steps) or down towards poorer health (4 possible steps). One of the nine possible steps was no change in SRH.

### Potential confounders

Age was categorized in < 49 years, 50–54 years, 55–59 years, 60–64 years, and 65–69 year intervals. Cohabitation status was dichotomised in living alone yes/no, self reported diseases: cancer, diabetes and metabolic disturbance (yes/no), use of general practitioner in past 3 months (yes/no), working: engagement in active employment (yes/no), being menopausal (yes/no), psychosocial working environment: too busy (no/yes) (*Are You often so busy that it is hard to get your tasks done?*) with the answering categories: never, rarely, sometimes, often, almost always. Tempo too high (yes/no) ("*How do You experience the rate and pressure of the workload"*) with the answering categories: far too high, a little too high, appropriate, a little to low, far too low. Influence on working day (yes/no) (*" How great an influence do You normally have on the organization of Your day at work"*) with the answering categories: great amount of influence, some amount of influence, a little amount of influence and no influence. Daily smoking (yes/no), Diet: daily consumption of fruit and vegetables (yes/no), physical activity more than 4 hours/week (yes/no), alcohol consumption > 5 units last weekend (yes/no).

### Statistical analysis

Data were analysed by means of multivariate logistic regression analysis with odds ratio (OR) and 95% confidence intervals. All important potential confounding factors with p-values < 0.25 in the univariate analyses were included in the multivariate analyses. In the multivariate analysis we made the analyses with all potential confounders and then excluded insignificant variables to reduce loss of individuals. Interaction between BMI-changes and selected variables (age, cohabitation status, smoking status and psychosocial working environment factors at baseline) were statistically tested by means of logistic regression analysis.

When examining our secondary hypothesis we wanted to examine the odds of gaining weight among women who rated their health as sub-optimal (fair, poor or very poor) at baseline. An increase or a decrease in SRH was still defined as a move, up towards better health (4 possible steps) or down towards poorer health (4 possible steps). Statistical analyses were performed using SAS version 9.1.

## Results

At entry, the mean age was 53.8 years (range 45 to 69 years, SD 6.47) and the mean height was 166 cm (range 130 cm to 191 cm, SD 5.63). Mean BMI increased from 23.6 kg/m^2 ^(range 13 – 65 kg/m^2^) in 1993 to BMI 24.4 kg/m^2 ^in 1999. During the study period, 7898 (57.7%) women maintained a normal weight, 2313 (16.9%) gained weight and 601 women (4.4%) lost weight according to the WHO BMI-categories. Almost 80% (n = 10,770) of the women stayed in the same BMI-category both years and 20% changed BMI-category during the study period. At baseline, 11,596 (85%) rated their health as very good or good. Fifty-nine percent experienced no change in SRH. Twenty-three percent of the women rated their health poorer and 18% rated their health better according to the nine steps scale of SRH.

### Description of the study population

The percentage distribution of women who rated their health better (increase) or worse (decrease) in the period 1993 to 1999 is showed in Table 1 [see Table 1]. More than half of the women (59%) experienced no change in SRH and more rated their health poorer than better during the study period. Twenty three percent experienced a decrease in SRH and 18% experienced an increase in SRH [see Table 1].

**Table 1:** Percentage distribution of changes in self-rated (SHR) by nine possible steps (n = 13,684)

**Number of steps of change in SRH in the period from 1993 to 1999**	**Changes in SRH from 1993 to 1999**	**N**	**Percent (%)**
-4	From very good SRH in 1993 to very poor SRH in 1999	2	0.01
-3	From very good SRH in 1993 to poorly SRH in 1999	36	0.3
	From good SRH in 1993 to very poor SRH in 1999		
-2	From very good SRH in 1993 to fair SRH in 1999	317	2.3
	From good SRH in 1993 to poor SRH in 1999		
	From fair SRH in 1993 to very poor SRH in 1999		
-1	From very good SRH in 1993 to good SRH in 1999	2790	20.3
	From good SRH in 1993 to fair SRH in 1999		
	From fair SRH in 1993 to poor SRH in 1999		
	From poor SRH in 1993 to very poor SRH in 1999		
0	No change in SRH	8102	59.2
1	From good SRH in 1993 to good very SRH in 1999	2266	16.5
	From fair SRH in 1993 to very poor SRH in 1999		
	From poor SRH in 1993 to fair SRH in 1999		
	From very poor SRH in 1993 to poor SRH in 1999		
2	From fair SRH in 1993 to very good SRH in 1999	155	1.1
	From poor SRH in 1993 to good SRH in 1999		
	From very poor SRH in 1993 to fair SRH in 1999		
3	From poor SRH in 1993 to very good SRH in 1999	15	0.1
	From very poor SRH in 1993 to good SRH in 1999		
4	From very poor SRH in 1993 to very good SRH in 1999	1	0.01

Total		13684	100.00

Table 2 shows baseline characteristics of the participants according to BMI categories in 1993 and in 1999 [see Table 2]. Individuals who were underweight in 1993 and who rated their health poorer during the study period were more likely to stay underweight in 1999 (61%).

**Table 2:** Baseline characteristics (%) in relation to changes in BMI (kg/m^2^) (n = 13,684)

Baseline characteristics (1993)	Underweight women in **1993 **(BMI <18.5) n = 309				Normal weight women in **1993 **(18.5 ≤ BMI < 25.0) n = 9676				Overweight women in **1993 **(BMI ≥ 25) n = 3699			
	Under-weight **1999**	Normal weight **1999**	Overweight **1999**	*n*	Under-weight **1999**	Normal weight **1999**	Overweight **1999**	*n*	Under-weight **1999**	Normal weight **1999**	Overweight **1999**	*n*

**Age**												
- 49	38	62	0	*106*	1	79	20	*3428*	0	7	93	*1013*
50–54	49	51	0	*67*	1	82	17	*2293*	0	10	90	*868*
55–59	57	43	0	*77*	1	84	15	*2039*	1	9	90	*907*
60–64	64	36	0	*36*	2	81	17	*1249*	1	13	86	*595*
65–69	78	22	0	*23*	1	86	13	*667*	1	13	86	*316*
**Cohabitation**												
Yes	49	51	0	*214*	1	82	17	*7449*	1	10	90	*2813*
No	56	44	0	*75*	1	80	19	*1886*	1	11	88	*750*
**Working**												
Yes	46	54	0	*226*	1	82	17	*7475*	1	9	90	*2695*
No	69	31	0	*74*	2	82	16	*1901*	1	12	87	*907*
**Smoking**												
No	49	51	0	*109*	1	82	17	*5715*	1	9	90	*2478*
Yes	52	48	0	*184*	2	81	16	*3522*	1	12	87	*1028*
**Physical activity**												
Yes	52	48	0	*278*	1	82	17	*9180*	1	10	89	*3389*
No	46	54	0	*28*	2	76	22	*410*	0	10	91	*267*

Ninety percent of the women who were normal weight in 1993, but overweight in 1993, rated their health poorer. Of the women who were underweight in 1993 and 1999, about half were smoking (51%). Among individuals who gained from normal weight to overweight, 22% compared to 76% of the women with stable normal weight, were not physically active [see Table 2]. Table 3 shows baseline characteristics (%) in relation to baseline SRH [see Table 3]. When we compare the women who rated their health as very good in 1993 with the women who rated their health very poor, we find that the women with poor SRH smoke more and exercise less.

**Table 3:** Baseline characteristics (%) in relation to baseline self-rated health (n = 13,684)

Baseline characteristics (1993)	**Self-Rated Health at base-line (N = 13684)**										
	Very good SRH (n = 5450)	*n*	Good SRH (n = 6146)	*n*	Fair SRH (n = 1833)	*n*	Poor SRH (n = 222)	*n*	Very poor SRH (n = 33)	*n*	

**Age**											
- 49	40	*2160*	32	*1941*	21	*388*	22	*48*	30	*10*	*4547*
50–54	24	*1299*	24	*1456*	22	*410*	25	*56*	21	*7*	*3228*
55–59	19	*1032*	23	*1385*	29	*520*	35	*77*	27	*9*	*3023*
60–64	12	*653*	14	*883*	17	*319*	9	*20*	15	*5*	*1880*
65–69	6	*306*	8	*481*	11	*196*	*9*	*21*	6	*2*	*1006*
**Self-rated health**											
Better (increase)	0	*0*	25	*1507*	41	*751*	*69*	*153*	79	*26*	*2437*
Unchanged	65	*3543*	58	*3556*	51	*931*	*30*	*65*	21	*7*	*8102*
Poorer (decrease)	35	*1907*	18	*1083*	8	*151*	*2*	*4*	0	*0*	*3145*
**Cohabitation**											
Yes	82	*4285*	79	*4701*	75	*1316*	*68*	*148*	81	*26*	*10476*
No	18	*972*	21	*1223*	26	*440*	*32*	*70*	19	*6*	*2711*
**Working**											
Yes	86	*4497*	79	*4720*	60	*1067*	*46*	*100*	38	*12*	*10396*
No	14	*789*	21	*1231*	40	*722*	*54*	*120*	62	*20*	*2882*
**Smoking**											
No	66	*3436*	64	*3737*	57	*997*	*56*	*118*	44	*14*	*8302*
Yes	34	*1761*	36	*2121*	43	*742*	*44*	*92*	56	*18*	*4734*
**Physical activity**											
Yes	97	*5230*	95	*5818*	90	*1617*	*77*	*164*	66	*21*	*12847*
No	3	*187*	5	*277*	10	*180*	*23*	*50*	34	*11*	*705*

### Association between SRH and weight changes

When women gained from underweight to normal weight, the odds showed a decrease in SRH (OR: 0. 59, 95% CI:0. 35–0.99) [see Table 4]. The odds of a decrease in SRH were higher among women who gained weight from normal weight to overweight OR: 1.18, 95%CI: 1.04–1.35). To lose weight from overweight in 1993 to normal weight in 1999 did not have an effect on health ratings (OR: 0.96, 95% CI: 0.76–1.23). The association between sub-optimal SRH at baseline (1993) and the odds of later weight gain (OR: 1.29, 95%CI: 1.10–1.51) is shown in Table 5 [see Table 5].

**Table 4:** Odds Ratio (OR) and 95% CI for a decrease in SRH by weight changes (n = 13,684)

**Changes in BMI categories from 1993 to 1999**	Underweight in 1999 (BMI <18.5 kg/m^2^)	Normal weight in 1999 (18.5 kg/m^2 ^≤ BMI < 25.0 kg/m^2^)	Overweight in 1999 (BMI ≥ 25 kg/m^2^)
Underweight in 1993 (BMI <18.5 kg/m^2^)	1.0 (reference)	0.59 (0.35–0.99)	---
Normal weight in 1993 (18.5 ≤ BMI < 25.0 kg/m^2^)	1.46 (0.93–2.29)	1.0 (reference)	1.18 (1.04–1.35)
Overweight in 1993 (BMI ≥ 25 kg/m^2^)	---	0.96 (0.76–1.23)	1.0 (reference)

* Adjusted for age, cohabitation status, diabetes, metabolic disturbance, use of General Practitioner last 3 months, engaged in active employment, menopause, smoking, diet (consumption of vegetables).

**Table 5:** Odds Ratio (OR) and 95% CI for weight gain by sub-optimal SRH at baseline (n = 9,012).

	**Odds Ratio (OR) and 95% CI for weight gain according to sub-optimal self-rated health at baseline**	**P-value**
**Self-rated health in 1993**		<0,0019
Optimal (very good or good)	1 (reference)	
Sub-optimal (fair, poor or very poor)	1,29 (1,10–1,51)	
**Age**		<0,0001
- 49 years	1 (reference)	
50–54 years	0,84 (0,73–0,97)	0,0141
55–59 years	0,67 (0,57–0,78)	<0,0001
60–64 years	0,80 (0,66–0,95)	0,0119
65–69 years	0,57 (0,44–0,73)	<0,0001
**Living arrangement**		0,0005
Cohabiting	1 (reference)	
Living alone	1,27 (1,11–1,45)	
**Use of general practitioner past 3 months**		0,0002
Less	1 (reference)	
More	0,81 (0,72–0,90)	

Women who lost weight were excluded from the analysis.

* Adjusted for age, living arrangements, diabetes, metabolic disturbance, use of General Practitioner last 3 months, engaged in active employment, menopause, psycho-social working environment (busyness), smoking, diet (consuming vegetables)

## Discussion

As a primary research question we hypothesised that weight changes were associated with changes in SRH (hypothesis 1). However, we anticipated that overweight women, who lost weight and became normal weight, would rate their health better than women who were overweight in both 1993 and 1999. We found that women who had been overweight and became normal weight, did not rate their health better than before and the women experienced a decrease in SRH, when they gained from normal weight in 1993 to overweight in 1999.

Only women, who were underweight at baseline and had become normal weight in 1999, experienced an increase in SRH during the study period. This group may consist of women getting well after being affected by illness.

As a secondary research question we wanted to examine if poor SRH at baseline lowered the odds of gaining weight due to underlying diseases (hypothesis 2). However, contrary to what we initially expected, we found that poor health at baseline increased the odds of later weight gain. Kristensen et al [6] have suggested this relation to be caused by a more unhealthy lifestyle among people who rate their health as poor. Manderbacka et al [25] found similar results when they investigated the association between lifestyle and SRH, with data from a face-to-face survey, among a sample representative of the Swedish population. One of the conclusions in that study was that poor life-style was associated with poor health. The results from the current study point toward the conclusion that weight stability is more beneficial to health ratings than any weight change. Also, a suboptimal SRH may be a predictor of later weight gain.

Several cross-sectional studies have found an association between obesity and SRH, and other related health measures, such as life satisfaction [8,26,27]. These studies find an association between obesity and poor SRH, or life satisfaction, and suggest that obesity may lead to poor SRH. However, none of the studies have examined the reversed causality. One study only examined the association between weight change and change in health-related life satisfaction [14]. They found that compared to remaining overweight, weight loss among the obese was associated with gain in health-related quality of life (HRQL) [14]. However, health-related quality of life and SRH may be two different measures, and our results may therefore not be comparable [28].

There is general agreement that SRH provides a useful summary of how individuals perceive their overall health status, including both physical and mental health. A large number of studies have consistently shown, in a wide range of disease areas, that SRH is a powerful predictor of clinical outcome and mortality [29-31]. A meta-analysis of 117 weight loss treatment tests showed that people who enter weight loss treatments (intentional) seem to emerge with less depression and greater self-esteem [12]. However, health-related quality of life and self-esteem are different concepts a different meaning to people, than the concept of SRH. We have found no other published results that were comparable to ours, e.g. that studied the association between weight changes and changes in SRH.

### Strengths and weaknesses

The present study is based on a large population of Danish female nurses (n = 19,898) with a high response rate at baseline (86%) and at follow-up (77%). The Danish Nurse Cohort Study consists of a homogeneous group in relation to several potential confounding factors. The nurses have the same sex, same education and only a small spread in age (44–69 years). Danish nurses exercise more, smoke less, are slimmer and rate their health better, than the general female population in Denmark [32], which may have resulted in an underestimation of the true associations. Potential confounding factors, exposure and outcome measures, are based on self-reported data, which raise a question regarding validity. It is well known that overweight women have a tendency to underreport their weight [33,34]. An American study estimated that 35% of all adult women underreported their weights [35]. Indeed, if the obese women systematically misinformed about their weight, a specific misclassification bias may have been a problem in the present study and if the nurses tended to underreport weight in 1999 compared to 1993, the effect on SRH will have been underestimated. Validation on weight measures in the Danish Nurse Cohort Study is still lacking. When discussing the association between weight changes and SRH, the intention behind the weight changes is an important issue. In some cases it may be assumed that unintentional weight loss would have a negative impact of SRH, as unintentional weight changes may be caused by diseases or life style changes such as lack of physical activity or change in diet. However, weight gain can also be a sign of regained health or of increased unhealthy behaviour. Also, weight loss can be caused by several reasons such as successful slimming efforts or change of life style or diseases. Studies have shown associations between depressed mood and weight gain [36]. When measuring changes in SRH the statistical problem "Regression towards mean" may occur and will increase the likelihood that women who rated their health very poor in 1993 would rate it less poor in 1999. In our study less than 2% (n = 255) of the women rated their health poor and very poor at baseline.

Furthermore, a "floor and ceiling effect" may have occurred in this study reducing the variation and the potential for finding associations even if present. This problem may have attenuated the results in this study. Unfortunately, useful information on intentional weight loss was not accessible. We controlled for several important diseases including use of general practitioner, and still found significant association. However, the risk of residual confounding by underlying diseases may still be a possibility.

## Conclusion

In a summery, we found an association between changes in BMI and changes in SRH. Women who were underweight in 1993 but normal weight in 1999, rated their health better than the women who were underweight throughout the study period. Women who were normal weight in 1993 but overweight in 1999 rated their health poorer than women who were normal weight both years. Surprisingly, women who were overweight in 1993 but normal weight in 1999, did not rate their health better than those who remained overweight. This could be due to diseases not controlled for or due to unintentional weight loss among the overweight and obese. There is a need for further research of the health consequences concerning weight changes. Health benefits or consequences of a stable overweight compared to a weight loss among overweight women are still unknown. It would be most essential for public health to examine this issue further as the obesity problem grows and more people than ever are trying to lose weight. More studies examining relations of intentional changes in body weight and health consequences are warranted.

## Competing interests

The authors declare that they have no competing interests.

## Authors' contributions

MKS carried out the analysis and drafted the manuscript. YAH and MG participated in the design of the study and data. BLH participated in the design of the study and assisted with the draft of the manuscript and the statistical analysis. All authors read and approved the final manuscript.

## Pre-publication history

The pre-publication history for this paper can be accessed here:


